# The Assessment of *CDX1, IHH, SHH, GATA4, FOXA2, FOXF1* in Congenital Intra-Abdominal Adhesions

**DOI:** 10.15388/Amed.2024.31.1.15

**Published:** 2024-02-27

**Authors:** Helēna Freijere Pope, Māra Pilmane, Anna Junga, Aigars Pētersons

**Affiliations:** 1 Institute of Anatomy and Anthropology, Riga Stradiņš University, Riga, Latvia; 2 Children’s Clinical University Hospital, Riga Stradiņš University, Riga, Latvia

**Keywords:** Congenital intra-abdominal adhesions, CDX1, IHH, GATA4, SHH, FOXA2, FOXF1, įgimtos vidinės pilvo sąaugos, CDX1, IHH, GATA4, SHH, FOXA2, FOXF1

## Abstract

Congenital abdominal adhesions are a rare condition that can result in a small bowel obstruction at any age, more frequently in pediatric populations. The cause remains unknown, and the importance of aberrant congenital bands is related to the difficulty of diagnosis, and cases of death with late detection have been documented. This research examines the expression of Caudal Type Homeobox 1 (CDX1), Indian Hedgehog (IHH), Sonic Hedgehog (SHH), GATA Binding Protein 4 (GATA4), Forkhead Box A2 (FOXA2) and Forkhead Box F1 (FOXF1) gene expression in human abdominal congenital adhesion fibroblast and endothelium cells by chromogenic in situ hybridization, with the aim of elucidating their potential association with the etiology of congenital intra-abdominal adhesion band development. The potential genes’ signals were examined using a semi-quantitative approach. Significant correlations were observed between the expression of CDX1 (*p* <.001) and SHH (*p*=0.032) genes in fibroblasts from congenital intra-abdominal adhesions compared to fibroblasts from control peritoneal tissue. Statistically significant very strong correlations were found between the CDX1 and IHH comparing endothelium and fibroblast cells in congenital abdominal adhesion bands. There was no statistically significant difference found in the distribution of IHH, FOXA2, GATA4, and FOXF1 between the fibroblasts and endothelium of the patients compared to the control group. The presence of notable distinctions and diverse associations suggests the potential involvement of numerous morpho-pathogenetic processes in the development of intraabdominal adhesions.

## Abstract (it)

Įgimtos pilvo sąaugos yra reta būklė, kuri gali sukelti plonosios žarnos nepraeinamumą bet kuriame amžiuje, tačiau dažniau pasitaiko vaikams. Priežastis vis dar nežinoma, o įgimtų aberacinių sąaugų svarba susijusi su diagnozavimo sunkumais, be to, užfiksuota mirties atvejų, kai jos buvo nustatytos vėlai. Šiame tyrime nagrinėjama Caudal Type Homeobox 1 (CDX1), Indian Hedgehog (IHH), Sonic Hedgehog (SHH), GATA Binding Protein 4 (GATA4), Forkhead Box A2 (FOXA2) ir Forkhead Box F1 (FOXF1) genų raiška žmogaus pilvo įgimtų vidinių adhezinių juostų fibroblastų ir endotelio ląstelėse chromogeninės in situ hibridizacijos metodu, siekiant išsiaiškinti jų galimą ryšį su įgimtų pilvo vidinių adhezinių juostų vystymosi etiologija. Potencialių genų signalai buvo tiriami taikant pusiau kiekybinį metodą. Nustatyta reikšminga koreliacija tarp CDX1 (p <,001) ir SHH (p=0,032) genų raiškos fibroblastuose, gautuose iš įgimtų pilvo vidaus adhezinių juostų, palyginti su fibroblastais, gautais iš kontrolinio pilvaplėvės audinio. Nustatytos statistiškai reikšmingos labai stiprios koreliacijos tarp *CDX1* ir *SHHH* endotelio ir fibroblastų ląstelių iš įgimtų pilvo organų adhezinių juostų. *IHH, FOXA2, GATA4* ir *FOXF1* pasiskirstymo tarp pacientų fibroblastų ir endotelio, palyginti su kontroline grupe, statistiškai reikšmingų skirtumų nenustatyta. Žymių skirtumų ir įvairių sąsajų buvimas rodo galimą daugelio morfopatogenetinių procesų dalyvavimą vystantis intraabdominalinėms adhezijoms.

## Introduction

Although very rare, a congenital adhesion band, also known as an aberrant congenital band, has the potential to result in small intestinal obstruction across all age groups, usually in children. However, there are only a limited number of isolated case reports available [1]. The incidence with which congenital abdominal adhesions lead to small intestinal obstruction is uncertain, as the underlying etiology of this phenomenon remains unidentified [2]. A congenital adhesion band refers to an intra-abdominal adhesion that is believed to have originated congenitally or de novo, without any association with intra-abdominal conditions such as inflammatory diseases or surgical procedures, or embryologic leftovers like the omphalomesenteric duct or vitelline artery remnants, according to current research [3].

The majority of congenital bands are often seen in the small intestine, whereas their occurrence in the large intestine is quite uncommon [4]. The challenge associated with diagnosing this condition is closely linked to the need of identifying aberrant congenital bands. Instances of mortality resulting from delayed identification have been documented [5].

When considering the molecular basis of intestinal differentiation, it is crucial to take into account the involvement of genes. Genes play a significant role in determining the fate of both the endodermal and mesodermal components of the gut [6]. Consequently, alterations in genes associated with intestinal development may potentially be responsible for the occurrence of congenital intraabdominal adhesions.

The *CDX* gene products are recognized to have a significant role in intestinal patterning, even though the molecular processes driving this process are not well understood [7]. The expression of murine and human *CDX1* appears to be restricted to the intestine and colon in mature organisms. The human *CDX1* gene was discovered in a small intestine cDNA library using a murine cDNA probe [8]. Using anti-*CDX1* antibodies, it has been demonstrated that the *CDX1* protein is localized in the proliferating embryonic epithelium of fetal murine intestinal tissues throughout development. However, in the adult intestine and during postnatal differentiation, it is restricted to the compartment of proliferative crypts. In the postnatal intestine and during embryonic development, the mesenchymal layer has been found to be completely negative for the *CDX1* gene [9]. Several differentiation markers, including villin and cytokeratin 20, have been shown to be transcriptionally controlled directly by *CDX1* [11]. While *CDX1* is expressed in the intestine around the middle of gestation, some studies indicate that it has no discernible effect on gastrointestinal development [10].

*GATA4* serves a crucial function in regulating early intestinal epithelial cell proliferation [12]. It is predominantly expressed in the gut’s proximal small intestinal epithelium. In fact, *GATA4* regulates specific gene networks along the anterior-caudal axis of the intestinal epithelium by activating jejunum-specific genes [13]. The midgut endoderm initially expresses *GATA4* during the early stages of gut development [13]. Although *GATA-4* is believed to be essential for the development of the rodent gastrointestinal mucosa, their role in the human digestive system remains unknown [14].

Multiple embryonic organs, including the developing intestines, require Hedgehog signaling for proper development [15]. In the mouse embryo, Sonic hedgehog (*SHH*) is expressed in endodermal epithelia from the oral cavity to the gastrointestinal tract, where it contributes to cell proliferation in the underlying mesenchyme and subsequent differentiation into the gastrointestinal smooth muscle [15]. Indian hedgehog (*IHH*) and Sonic hedgehog are two hedgehog genes with distinct, partially overlapping expression patterns. Sonic hedgehog is only expressed in the oral, tracheal, lung, and esophageal epithelia, whereas Indian hedgehog is expressed throughout the epithelium of the digestive passages extending from the stomach distally [15]. Mice lacking the Indian hedgehog have diminished intestinal intervillous epithelium proliferation [16], but current data about the humans are incomplete.

Many tissues arising from endoderm depends on the Forkhead box A (*FOXA*) family of pioneer transcription factors for their development [17]. During gastrulation *FOXA2* is crucial for the development of the definitive endoderm as well as the differentiation of tissues generated from the endoderm [18]. *FOXA2* are proven in studies to be critical regulators of goblet cell development and may also contribute to intestinal health maintenance [17]. It has been demonstrated that *FOXA2* also controls the expression of certain genes in the gut, such as the apolipoprotein B locus, mucins that form gels, and the transcription factors Pax6 and *HNF6* [19]. Peak *FOXA2* occupancy was frequently seen in loci involved [17]. Additionally, the *FOXA* families of transcription factors could collaborate to control gene expression across the whole genome in the intestinal epithelium [18]. Therefore, this gene could be of interest also in intra- congenital adhesion development.

*FOXF1* and -2, mice’s Forkhead transcription factor genes, are expressed in splanchnic mesoderm derivatives, organs derived from the primordial gut. *FOXF1* is extensively expressed in the developing gut [20]. *FOXF1* plays a crucial role in the division of the lateral plate into two distinct components: the splanchnic and somatic layers [21]. The inability to assess the function of *FOXF1* during later stages of gut development is hindered by the fact that null mutants exhibit embryonic mortality [22]. *FOXF1* gene mutation heterozygotes have high neonatal mortality rates, which can exceed 90% on some genetic backgrounds, due to lung and foregut abnormalities. The *FOXF1* mutant has reduced cellular adhesion. Mutants exhibit a ganglionic megacolon with smooth muscle hypoplasia, occasional anal atresia, club-shaped villi, and multilayered epithelia [23].

Based on the above mentioned, the objective was to investigate the expression of *CDX1, IHH, SHH, GATA4, FOXF1, FOXA1* genes, and to identify any potential associations among these genes in the congenital intra-abdominal adhesions.

## Materials and Methods

### Information about the Patients

This study was conducted in accordance with the 1964 Declaration of Helsinki, and the study protocol was approved by the Ethical Committee of Riga Stradiņš University on 10th of May 2007. Following a comprehensive description of the research, written consent was procured from the parents or legal guardians of all participating patients.

The study group consisted of 14 children (5 males and 9 females) diagnosed with congenital intra-abdominal adhesions. The inclusion criteria were as follows: diagnosed with congenital intra-abdominal adhesion band. The exclusion criteria were intra-abdominal inflammation or any other pathology except congenital adhesion band formation. Patients included in the study ranged in age from 1 to 134 days and had intra-abdominal adhesions in various locations, including Ladd’s band, the duodenum, the jejunum, and the proximal and distal ileum (Table 1). Also, each patient’s diagnosis varied (Table 1). Chromosomal abnormalities or monogenic syndromes were not tested by cytogenetic and whole exome testing in these patients.

**Table 1:** Information about the patients.

No.	Gender	Age (days)	Adhesion location	Diagnosis
1	F	1	LB	Left sided diaphragmatic hernia, Ladd’s band
2	F	1	LB	Meconium ileus, Ladd’s band, intestinal malrotation
3	M	2	D	Ileal atresia
4	M	2	JI	Intrauterine ileus with ileum distal part volvulus, caecum necrosis. Ileostomy
5	M	3	LB	Intestinal malrotation, small bowel volvulus (360°) with poor circulation in ileum part (16 cm), Ladd’s band. Ileostomy
6	M	4	D	Intestinal malrotation, partial intestinal obstruction, congenital intra-abdominal adhesion band in duodenum part
8	F	9	D	Gastroschisis
9	M	14	JI	Gastroschisis. State after gastroschisis repair surgery. Ileostomy
10	F	51	LB	Intestinal malrotation, congenital intra-abdominal adhesion bands. Ladd’s band
11	F	56	JI	Gastroschisis. State after gastroschisis repair surgery. Ileostomy
12	F	71	JI	Small bowel volvulus (360°), condition after partial intestinal obstruction. Ladd’s band division
13	F	94	JI	Partial intestinal obstruction. State after diaphragmatic hernia repair surgery
14	F	134	JI	Partial intestinal obstruction. State after mesenteric thrombosis, partial jejunum, total ileocecal angle and colon ascendens resection, jejuno-trasversostomy forming and closing

Abbreviations: LB – Ladd’s band; D – duodenum; JI – jejunum and ileum proximal part; DI – ileum distal part; F – female, M – male

The control group consisted of 4 males and 2 females, aged 46–92 days. Samples of peritoneal tissues were obtained during right and left side inguinal hernia repair surgeries. The inclusion criteria were diagnosed with right or left side hernia and no congenital intra-abdominal adhesion bands in anamnesis or family history. The exclusion criteria were inflammation or any other pathology present.

### Chromogenic In Situ Hybridization

The decision was made to employ chromogenic in situ hybridization (CISH) as a technique for seeing the putative messenger RNA (mRNA) transcripts of the candidate genes [24].

Tissue specimens were obtained and afterwards immersed in a solution consisting of 2% formaldehyde, 0.2% picric acid, and 0.1M phosphate buffer (pH 7.2) for a duration of one day. Subsequently, the samples underwent a rinsing process in Tyrode’s buffer, which consisted of NaCl, KCl, CaCl2•2H2O, MgCl2•6H2O, NaHCO3, NaH2PO4•H2O, and glucose, containing 10% saccharose. The rinsing process was conducted for a duration of 12 hours, after which the samples were embedded in paraffin.

The tissue samples were appropriately documented and assigned randomized codes. In addition, the researchers and laboratory assistants had access only to the patients’ history (Table 1) and no additional relevant information was provided.

In the study *CDX1, IHH, SHH, GATA4, FOXA2, FOXF1* probes were used: Caudal Type Homeo box 1 (CDX1-20-DIG, 5q32, Empire Genomics, New York, USA), Indian Hedgehog (IHH-20-DIG, 2q35, Empire Genomics, New York, USA), Sonic Hedgehog (SHH-20-DIG, 7q36.3, Empire Genomics, New York, USA), GATA Binding Protein 4 (GATA4-20-DIG, 8p23.1, Empire Genomics, New York, USA), Forkhead Box A2 (FOXA2-20-DIG, 20p11.21, Empire Genomics, New York, USA), Forkhead Box F1 (FOXF1-20-DIG, 16q24.1, Empire Genomics, New York, USA).

Probe *CDX1, SHH, IHH, GATA4, FOXA2* and *FOXF1* sets consisted of DNA labeled in Digoxigenin. The DNA probe sets were specifically designed to form hybridization bonds with specific chromosomal regions. These regions include *CDX1* with the chromosomal region 5q32, *SHH* with the chromosomal region 7q36.6, *IHH* with the chromosomal region 2q35, *GATA4* with the chromosomal region 8q23.1, *FOXA2* with the chromosomal region 20q11.21, and *FOXF1* with the chromosomal region 16q24.1. The hybridization was performed on both normal metaphase spreads and interphase nuclei.

The pretreatment process was conducted in accordance with established laboratory protocols. The process of denaturation and hybridization started by carefully dispensing 10 microliters of the probe onto each pretreatment specimen with a pipette. Subsequently, the specimens were affixed with an 18 × 18 mm coverslip and positioned onto a hot plate set at a temperature of 79 oC for a duration of 5 minutes. Subsequently, the specimens were relocated to a controlled humidity room and subjected to overnight hybridization at a temperature of 37 oC, with measures taken to prevent desiccation. On the subsequent day, the slides were immersed in SSC wash buffer followed by TBS wash buffer in order to eliminate the coverslips. Additionally, the specimens were subjected to the subsequent stages of the chromogenic in situ hybridization (CISH) method in accordance with the manufacturer’s instructions. The slides were moved into a staining jar and afterwards rinsed under a stream of cold running water for a duration of 2 minutes. Following this, the slides were subjected to dehydration using 100% ethanol and subsequently incubated in xylene.

To prevent the occurrence of trapped air bubbles, the coverslips were carefully reattached, after which the specimens were subjected to analysis using a light microscope. The turquoise-colored dots were used to show the specific gene area that was targeted, whereas the bright red hue was employed to represent the control group. The hybridized probe fragments were seen following the application of a nuclear dye for counterstaining the nucleus. During the interphase of normal cells or cells without abnormalities, it was anticipated that two separate dots would develop within the nucleus of the cells.

A semi-quantitative scoring method was used to conduct the examination of the specimens [25]. The evaluation of the outcomes was conducted by the assessment of turquoise-colored dots observed in a minimum of five randomly chosen fields of vision at a magnification of 1000×, employing immersion oil. The copies obtained from the turquoise probes were assessed in the fibroblasts and endothelium derived from the collected samples.

Structures were labelled as follows: 0, no turquoise copies detected (0%); 0/+, occasional turquoise copies detected (12.5%); +, few copies detected (25%); +/++, few to moderate copies detected (37.5%); ++, moderate number of turquoise copies detected (50%); ++/+++, moderate to numerous copies detected (62.5%); +++, numerous turquoise copies detected in the visual field (75%); +++/++++, more than numerous copies detected in the visual field (87.15%), ++++ (100%) all cellular entities demonstrate the expression of turquoise copies [25].

For visual illustration, Leica LEITZ DM RB microscope, Euromex Scientific Camera DC.20000i, and the image processing and analysis software ImageFocusAlpha (Euromex Microscopen bv, Arnhem, The Netherlands) were used.

### Statistical Analysis

Jamovi 2.3.28 (The jamovi project (2023). *jamovi* (Version 2.3) [Computer Software]) was used for data analyses. The semi-quantitative evaluation findings were converted into numerical values. For instance, a score of 0 was assigned to indicate 0, a score of 1 was assigned to indicate 0/+, and a score of 2 was assigned to indicate +, score of 3 was assigned to indicate +/++, score 4 was assigned to indicate ++, a score of 5 was assigned to indicate ++/+++, and a score of 6 was assigned to indicate +++, a score 7 was assigned to indicate +++/++++, a score 8 was assigned to indicate ++++.

## Results

The presence of genes, as turquoise-colored dots, was observed in the minority of patient sample cases, however, there were high gene-mRNA copy *CDX1* containing cells in control group patients (Table 2). Overall, gene-mRNA-copy-containing cells were observed more in fibroblasts than endothelium. The presence of *CDX1* in the fibroblasts and endothelium of congenital intra-abdominal adhesion band samples exhibited a range of results, with some samples showing no copies (0) detected and others showing occasional (0/+) gene copies detected (Table 2).

**Table 2:** The relative number of *CDX1, IHH, SHH, GATA4, FOXA2, FOXF1* gene copies in the congenital intra-abdominal adhesion fibroblasts and endothelium and in the control group

Congenital intra-abdominal adhesion band						
**Patient’s No.**	**Fibroblasts**			**Endothelium**		
	***CDX1***	***IHH***	***SHH***	***CDX1***	***IHH***	***SHH***
1	0	0/+	0	0	0	0
2	0	0	0	0	0	0
3	0	0	0	0	0	0
4	0	+	0	0	0	0
5	0	0/+	0	0	0	0
6	0	0	0	0	0	0
7	0/+	0	0	0/+	0	0
8	0/+	0/+	0	0	0	0
9	0/+	0	0	0	0	0
10	0	++	0	0	0/+	0
11	0	0	0	0	0	0
12	0	0	0	0	0	0
13	0	0	0	0	0	0
14	0	0	0	0	0	0
Control group No.	**Fibroblasts**			**Endothelium**		
	***CDX1***	***IHH***	***SHH***	***CDX1***	***IHH***	***SHH***
1	+++	0	0	++	0	0
2	+++	0	0	++	0	0
3	0	0	0	0	0	0
4	+	0	0/+	0/+	0	0
5	+++	0	0/+	++	0	0
6	+++	0	0	++	0	0

Abbreviations: No.—patient’s number, CDX1— Caudal Type Homeobox 1, IHH—Indian Hedgehog, SHH—Sonic Hedgehog.

**Figure 1:**
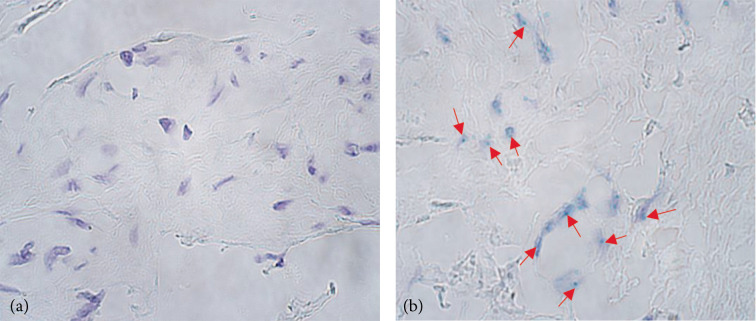
The chromogenic in situ hybridization micrographs of *CDX1* in a congenital adhesion band and a control patient were captured at a magnification of 1000×, utilizing immersion oil. (a) No gene copies (0) were found in congenital adhesion band sample fibroblasts or endothelium in 56 days old patient. (b) Numerous (+++) gene copies in the control group fibroblasts of a 92 days old child marked by arrows.

In contrast, the presence of the *CDX1* gene in the fibroblasts of the control group showed significant variability, ranging from no copies (0) detected to numerous gene copies (+++), with the majority of cases (4 out of 6) falling into the latter category. Similarly, the presence of the *CDX1* gene in the endothelium of the control group varied from no gene copies (0) to moderate (++) gene copies in most cases (4 out of 6), (Table 2, Figure 1).

**Figure 2:**
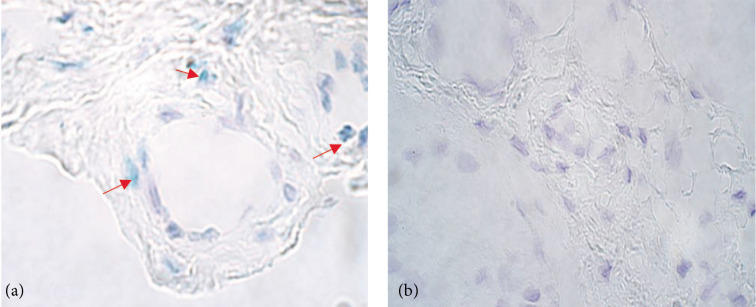
Chromogenic in situ hybridization micrographs of *IHH* in congenital adhesion band samples and control subject at 1000× magnification, using immersion oil. a) Moderate (++) gene copies in congenital adhesion band fibroblasts and occasional (0/+) gene copies of congenital adhesions band endothelium in a 135-day old patient marked by arrows. (b) No (0) gene copies in the control group endothelium of fibroblasts of a 56-day old child.

*IHH* presence in the fibroblasts and endothelium of congenital intra-abdominal adhesion band sample varied from no copies (0) detected to moderate (++) gene copies detected, however *IHH* gene presence in control group fibroblasts and endothelium had no copies (0) detected (Table 2, Figure 2).

**Figure 3:**
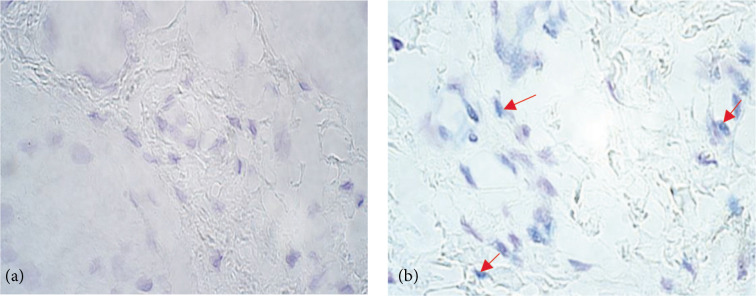
Chromogenic in situ hybridization micrographs of *SHH* in congenital adhesion band samples and control subject at 1000× magnification, using immersion oil. (a) No (0) gene copies in congenital adhesion band sample fibroblasts and endothelium of a 56-day old patient. (b) Occasional (0/+) gene copies in the control group fibroblasts of a 92-day old child.

The presence of *SHH* in the fibroblasts and endothelium of congenital intra-abdominal adhesion band samples exhibited a range of results, with some samples showing no copies (0) identified while others showed occasional (0/+) gene copies found. In contrast, the fibroblasts and endothelium of the control group did not exhibit any gene (0) copies detected (Table 2, Figure 3). The presence of *GATA4, FOXA2*, and *FOXF1* gene copies was not seen in the samples (0) of congenital intra-abdominal adhesion bands from patients, as well as in the control group (Table 2).

Statistical analysis involved only those genes which could be statistically analyzed, genes like *GATA4, FOXA2, FOXF2* and *SHH* in endothelium had sample size that low which couldn’t be analyzed.

A statistically significant difference was identified in the distribution of the *CDX1* gene between fibroblasts (p value 0.002) and endothelium (p value <.001) when comparing samples of congenital intra-abdominal adhesions from patients and control subjects. A statistically significant difference was identified in the expression of the *SHH* gene in fibroblasts (p = 0.032) when compared to the control group, as shown in Table 3. Moreover, in all of the examined samples, the p-values ranged from less than .001 to 0.585 (Table 3).

**Table 3:** The statistical importance of the distribution of *CDX1, IHH* and *SHH* genes between patients’ congenital intra-abdominal adhesion sample compared to control group.

Congenital intra-abdominal adhesion band sample vs. Control					
	Fibroblasts			Endothelium	
	*CDX1*	*IHH*	*SHH*	*CDX1*	*IHH*
Mann–Whitney U	8,5	27	28	8	39
p-Value	0.002	0.115	0,032	<.001	0.585
Effect size	0.7976	0.3571	0.3333	0.8095	0.0714

Abbreviations: vs.—versus, CDX1— Caudal Type Homeo box 1, IHH—Indian Hedgehog, SHH—Sonic Hedgehog.

A highly significant statistical connection was established between *CDX1* expression in fibroblasts and *CDX1* expression in endothelium, as evidenced by a strong Spearman’s correlation coefficient of 0.990. A significant link was detected between the expression of *IHH* in fibroblasts and *IHH* in endothelial cells, with a correlation coefficient (R value) of 0.837 (Table 4).

**Table 4:** The statistical correlation of CDX1 copies in the fibroblasts and endothelium, and of IHH copies in the fibroblasts and endothelium.

Factor 1	Factor 2	R	p-Value	Correlation
*CDX1* in fibroblasts	*CDX1* in endothelium	0.990	<.001	Very strong
*IHH* in fibroblasts	*IHH* in endothelium	0.837	<.001	Very strong

Abbreviations: CDX1— Caudal Type Homeo box 1, IHH—Indian Hedgehog.

## Discussion

Congenital intra-abdominal adhesion bands have received relatively less attention in studies pertaining to their developmental sources and processes, as compared to the majority of other congenital abnormalities [1]. This phenomenon can be attributed, in part, to its relatively low prevalence in comparison to other congenital abnormalities. The etiology of this condition remains poorly known and has not yet been thoroughly investigated, especially in humans [26].

Genetic factors exert a substantial influence on the developmental outcomes of both the endodermal and mesodermal components of the gastrointestinal tract, therefore, it is possible that changes in genes related to the development of the intestines might be accountable for the presence of congenital intra-abdominal adhesions [6]. The objective of this study was to analyze and measure gene expressions in fibroblasts and endothelial cells in newborns with congenital intra-abdominal adhesion bands. This investigation aimed to enhance our comprehension of the diverse functions of gene expression and its potential involvement in the development of this pathological condition.

Statistically significant variations were seen in the number of *CDX1* positive cells when comparing control fibroblasts to patient fibroblasts, as well as control endothelium to the endothelium of patients with intra-abdominal adhesions. The high expression of the *CDX1* gene was observed in both control fibroblasts and endothelium. However, there were only limited instances of *CDX1* gene expression detected in the fibroblasts of patient’s congenital intra-abdominal band tissues. *CDX1* (the gene encoding *CDX1* protein) expression has been previously described in gastrointestinal development, *CDX1* is a transcription factor that modulates multiple processes, including cell proliferation, apoptosis, cell adhesion, and columnar morphology in gut mice models [27]. The transcription factor caudal-related homeobox protein 1 (*CDX1*) is essential for the development, differentiation, and homeostasis of the intestine [28]. *CDX1* is not expressed in the early definitive gut endoderm but appears at post somite stages just prior to the transition of the multilayered intestinal endoderm to a single-layered intestinal epithelium at 14 days post coitum [29]. The absence of *CDX1* in congenital intra-abdominal adhesions bands which usually would be found in gastrointestinal development could indicate a possible involvement and interaction of *CDX1* absence and with the formation of congenital intra-abdominal adhesions bands. There has been research conducted that demonstrate how the loss of *CDX1* function is implicated in tumor genesis [28], and studies indicating that *CDX1* exerts an inhibitory effect on cellular proliferation by impeding the passage of cells through the G1 phase of the cell cycle [28], which could also indicate that the loss of *CDX1* could cause pathological hyperproliferation of fibroblasts and abdominal adhesion band formation. However, there are also studies that suggest that the absence of *CDX1* deletion does not have any discernible impact on midgut differentiation [30].

In our study significant statistical differences were seen in the quantity of *SHH* positive cells when comparing control fibroblasts to patient fibroblasts, but no statistical differences when comparing control endothelium to the endothelium of patients with intra-abdominal adhesions. Furthermore, it is crucial to acknowledge that there were observed instances of downregulation of the *SHH* gene in our study. The patients demonstrated no expression of the *SHH* gene in fibroblasts of congenital intra-abdominal adhesion tissues in comparison to the control group which did display expression of the *SHH* gene in some fibroblast cells. The potential association between Sonic hedgehog’s participation in the development of congenital intra-abdominal adhesions is plausible due to its regulatory function in intestinal development [31]. The proteins known as *IHH* and *SHH* have been recognized for their significant involvement in the processes of intestinal cell proliferation and differentiation. The hypoproliferation of the intestinal epithelium occurs as a consequence of the inhibition of these mechanisms [32]. Sonic hedgehog (*SHH*) expression may be observed in the endodermal epithelia spanning from the oral cavity to the gastrointestinal tract in mouse embryos [15]. In studies mice with a deficiency in the Sonic hedgehog gene display a range of gastrointestinal abnormalities, such as tracheo-esophageal fistula and anorectal atresia. These anomalies are frequently observed in people with mutations in genes related to the hedgehog signaling system and are considered common congenital gastrointestinal malformations [16]. The presence of a heterozygous mutant mouse for *SHH* deletion results in a range of gastrointestinal abnormalities, including the transformation of the stomach into intestinal tissue, the occurrence of annular pancreas, and the narrowing of the duodenum [16]. Also the stimulation of the Sonic hedgehog pathway is related with the intestinal stem cell activity in a rat model. The inhibition of cell proliferation was shown to be connected with the inhibition of the Sonic hedgehog signaling cascade [33].

Indian hedgehog is important factor of intestinal development; therefore, it could be also involved into pathogenesis of congenital intra-abdominal adhesion formation. The intestinal epithelial *IHH* communicates with the mesenchymal compartment in order to control the development and growth of mesenchymal cells, which subsequently impacts the proliferation and differentiation of epithelial cells [34]. In our study no significant statistical differences were seen in the quantity of *IHH* positive cells when comparing control fibroblasts to patient fibroblasts and no statistical differences when comparing control endothelium to the endothelium of patients with intra-abdominal adhesions. Furthermore, it is crucial to acknowledge that there were observed instances of upregulation of the *IHH* gene in our study. The patients demonstrated expression of the *IHH* gene in fibroblasts in comparison to the control group, but the control group tissues did not display any expression of the *IHH* gene in fibroblast tissues. But a noteworthy association was observed between the expression of *IHH* in fibroblasts and *IHH* in endothelial cells, exhibiting a robust correlation coefficient in congenital intra-abdominal adhesion tissue. The Sonic hedgehog’s involvement with the formation of congenital intra-abdominal adhesions could not be excluded because of its regulatory role during intestinal development. Studies have been made that show – at 16.5 days post-conception – that the epithelium of mouse embryos with intact *IHH* genes exhibits a monolayer of polarized epithelial cells, which are arranged in crypt-like structures. On the other hand, the colonic epithelium of embryos without the Indian hedgehog gene (*IHH*−/−) has a multilayered structure and does not exhibit the characteristic organization into crypts [35]. There have been reports that indicate the disruption of the intestinal mesenchymal architecture as a result of the Indian hedgehog gene deletion like the absence of the muscularis mucosae, degradation of the extracellular matrix, and a decrease in the population of crypt myofibroblasts [36].

It is noteworthy that there were no statistically significant disparities seen in the presence of cells expressing the *FOXA2, GATA4*, and *FOXF1* genes within the fibroblasts or endothelium when comparing tissues from the control group to those from the patient group. No expression of these genes was seen in either the control group or patient group. The potential participation of genes *FOXA2, GATA4*, and *FOXF1* in the establishment of abdominal adhesion bands cannot be disregarded due to their regulatory function in intestinal development [13,15,17].

A primary limitation pertains to the sample size of both the patient group and the control group, potentially exerting an influence on the outcomes. The limited accessibility of tissue material is mostly attributed to ethical considerations surrounding its harvesting. Several other factors also could limit this research, such as alterations in tissue gene expression associated with age, modifications in protein expression and localization resulting from tissue development in older children, may potentially influence the results. In the conducted study, the sample population encompassed individuals ranging from 1 to 134 days of age, hence introducing potential implications for the obtained outcomes.

Another limitation of this particular investigation pertains to the exclusive utilization of CISH as the method for detecting *CDX1, IHH, SHH, GATA4, FOXA2*, and *FOXF1* proteins inside the control and congenital intra-abdominal adhesion tissue cohorts. The use of other techniques such as immunochemistry has the potential to provide further insights and data for this investigation.

The assessment of congenital abdominal adhesion affected tissue can provide a description of the features and details of the growth and development of this tissue after birth. However, it is unable to provide a comprehensive understanding of the morphopathogenetic alterations that have taken place during the prenatal period.

Furthermore, it is important to note that this study is subject to several limitations, namely the scarcity of existing research on the development of the digestive tract and the specific genes that are expressed throughout this process. Typically, the data collection process mostly relied on animal models, so constraining the extent of our study database.

## Conclusions

The observed increase in *CDX1* gene expression in the fibroblasts and endothelium of the control group, as opposed to the afflicted tissue of congenital intra-abdominal adhesions, indicate the relation stimulation of developed congenital intra-abdominal bands.

Also, the presence of a statistically significant elevation of *SHH*-containing cells in the fibroblasts of the control group, in comparison to the affected tissue of individuals with congenital intra-abdominal adhesions, suggests a potential involvement of *SHH* in the persistence of congenital intra-abdominal adhesions.

Finally, presence of *IHH* in some patients’ adhesion fibroblasts only proves the existing dysregulation of *SHH, IHH* appearance and stimulates the adhesion development.
